# Alexithymia in Gastroenterology and Hepatology: A Systematic Review

**DOI:** 10.3389/fpsyg.2018.00470

**Published:** 2018-04-06

**Authors:** Danilo Carrozzino, Piero Porcelli

**Affiliations:** ^1^Department of Psychological, Health and Territorial Sciences, University “G.d'Annunzio” of Chieti-Pescara, Chieti, Italy; ^2^Psychiatric Research Unit, Psychiatric Centre North Zealand, Copenhagen University Hospital, Hillerød, Denmark

**Keywords:** alexithymia, gastroenterology, hepatology, somatization, Toronto Alexithymia Scale

## Abstract

**Background:** Alexithymia is a multifaceted personality construct that represents a deficit in the cognitive processing of emotions and is currently understood to be related to a variety of medical and psychiatric conditions. The present review aims to investigate the relationship of alexithymia with gastrointestinal (GI) disorders as functional gastrointestinal disorders (FGID, as irritable bowel syndrome (IBS) and functional dyspepsia) and inflammatory bowel disease (IBD) [ulcerative colitis (UC) and Crohn's disease (CD)] and liver diseases as chronic hepatitis C (CHC), cirrhosis, and liver transplantation.

**Methods:** The articles were selected from the main electronic databases (PsycInfo, Medline, PubMed, Web of Science, Scopus, Cochrane, and ScienceDirect) using multiple combinations of relevant search terms (defined GI and liver diseases, articles in English, use of the Toronto scales [TAS] for alexithymia). The TAS was selected as inclusion criterion because it is the most widely used measure, thus allowing comparisons across studies.

**Results:** Forty-eight studies met the inclusion criteria, of which 38 focused on GI disorders (27 on FGID and 11 on IBD) and 10 on liver diseases. Most studies (*n* = 30, 62%) were cross-sectional. The prevalence of alexithymia was higher in FGID (two third or more) than IBD and liver diseases (from one third to 50% of patients, consistent with other chronic non-GI diseases) than general population (10–15%). In functional disorders, alexithymia may be viewed as a primary driver for higher visceral perception, symptom reporting, health care use, symptom persistence, and negative treatment outcomes. Also, it has been found associated with psychological distress and specific GI-related forms of anxiety in predicting symptom severity as well as post-treatment outcomes and is associated with several psychological factors increasing the burden of disease and impairing levels of quality of life. A number of critical issues (small sample sizes, patients referred to secondary and tertiary care centers, cross-sectional study design, use of one single scale for alexithymia) constitutes a limitation to the generalization of findings.

**Conclusions:** Alexithymia showed to play different roles in gastroenterology according to the clinical characteristics and the psychological burden of the various disorders, with main relevance in increasing subjective symptom perception and affecting negatively post-treatment outcomes.

## Introduction

It is widely recognized that individual health status is only partially explained by the presence of a medically diagnosable disease. Subjective perception, thoughts, emotions, and behaviors associated with one's somatic status are sometimes clinically or more important than the medical diagnosis. Overall, the more the individual perception and behaviors are discrepant from those expected by medical recommendations, the less the course of disease (onset, recognition, presentation, maintenance, and outcomes) can be explained solely by biomedical factors. As documented by a large body of literature, psychosocial factors play a significant role in this regard. In one word, health and disease can be conceptualized and understood best within a biopsychosocial approach. Within this biopsychosocial perspective, each clinical factor is variously inter-connected with the others and has its own specific relative weight (Engel, 1980, 1982; Lipowski, 1984; Fava and Sonino, 2017).

Several authors attempted to explain the complex relationship between the individual illness experience and specific psychological factors when going beyond the misleading dichotomy between functional syndromes and organic diseases. For example, Pilowsky (1993, p. 62) defined abnormal illness behavior as “the persistence of a maladaptive mode of perceiving, experiencing, evaluating, and responding to one's health status, despite the fact that a doctor has provided a lucid and accurate appraisal of the situation and management to be followed.” Also, Lipowski's (1970) identified the most frequent meanings given by individuals to their illness (challenge, enemy, punishment, weakness, relief, strategy in interpersonal relations, irreparable loss or damage, and value). These subjective meanings can influence the likelihood to adequately recognize an underlying disease and to promptly seek medical care. Moreover, according to the self-regulatory model of Leventhal et al. (1980), subjective representations of illness determine the coping behaviors adopted and, consequently, the illness outcome. If a certain disease is considered unavoidable and dependent on factors outside of personal control (e.g., inflammation process, viral infection), it is unlikely that preventive measures (e.g., healthy lifestyle) will be adopted. More recently, the DSM-5 (American Psychiatric Association, 2013) dropped the “medically unexplained” criterion from the former diagnostic category of somatoform disorder of the DSM-IV and included positive psychological features (health anxiety, symptom preoccupation, and maladaptive illness behavior) in the new category of somatic symptom disorder. Finally, a recent international consensus recognized the value of patient-reported outcome (PRO) in order to inform shared decision making, clinical guidelines, and health policy. This international consensus provided indeed guidelines for PRO-specific information that should be included in clinical trial protocols (Calvert et al., 2018).

The strict neurohumoral interactions between the brain and the digestive system (referred to as the “brain-gut axis”) constitute a continuous bidirectional signaling homeostatic information system. Within this framework, gastrointestinal (GI) disorders are considered as one of the main bodily systems involved in the perception of somatic health. Gut sensations and symptoms emerge as a complex psychobiological process, whereby bottom-up inputs are modulated by top-down cognitive and emotional brain circuits (Van Oudenhove et al., 2016). GI illnesses are traditionally classified according to the biomedical model where causes and symptoms are specifically circumscribed to local organs and pathophysiological patterns. However, a wide body of research, as well as consolidated clinical experience, indicate that psychological factors significantly and negatively influence many clinical aspects such as the individual perception of gut sensations, the reported GI symptoms, as well as other clinically relevant illness-related behaviors. These findings have been reported in many GI conditions, from functional GI disorders (FGID) such as functional dyspepsia (FD) and irritable bowel syndrome (IBS) to chronic diseases such as inflammatory bowel disease (IBD) and liver diseases such as chronic hepatitis C (CHC). Not by chance, the term *hypochondriasis* derives etymologically from the Greek *hypokhondrios* (litteraly, “under the sternum”) because the viscera were considered the seat of melancholy.

FGID are functional multidetermined disorders arising from the gut mucosa, microbiota, and GI motility and FGID are caused by dysregulation of the brain-gut axis, with alterations at different levels of the enteric, autonomic and/or central nervous systems or a disturbed interplay between these systems (Ohman and Simrén, 2010). FGID are best understood from a biopsychosocial perspective. Evidence strongly suggest that: (1) there is no single biomarker for a comprehensive diagnosis of FGID, (2) compared to other GI disorders, psychiatric morbidity is more common (40–60%) among patients with FGID, (3) psychological and pharmacological treatments are only partially effective particularly on the long run, and a wax-and-wane temporal course characterized FGID, (4) psychological stress is one of the main triggers for onset and persistence of FGID (Drossman, 2016).

IBD are a group of chronic inflammatory intestinal diseases, the most common forms of which are ulcerative colitis (UC) and Crohn's disease (CD) whose pathophysiology is currently not fully understood. IBD is characterized by intermittent phases of quiescence and unforeseeable acute relapses. IBS and IBD are distinct entities, even though they do share some clinically relevant similarities. Symptom onset and clinical relapses in both IBS and IBD involve multifactorial, yet incompletely understood, triggers that likely include variable combinations of environmental, psychological and biological components such as altered gut microbiota and different grades of pro-inflammatory and immune activation (Spiller and Major, 2016). It is estimated that up to 60% of UC patients and more than one third of CD patients show symptoms that are common to IBS and IBD relapses such as abdominal pain, bloating, diarrhea, and loose stools. These overlapping IBD-IBS symptoms represent a source of considerable psychological distress for IBD patients, as well as a diagnostic and therapeutic conundrum for the physician (Stanisic and Quigley, 2014; Carter et al., 2017). Furthermore, there is consistent evidence of high prevalence of psychological disturbances (76% for anxiety and 41% for depression) during the acute phase of IBD (Fukuba et al., 2014; Neuendorf et al., 2016). Also, it was found that these psychological comorbidities had a bidirectional association with symptom activity (Sexton et al., 2017) and may predict acute relapses (Mikocka-Walus et al., 2016).

Of interest, IBD is not the only GI organic disease where psychological factors play a relevant role in explaining symptom reporting. Hepatology is a branch of gastroenterology in which patient-related outcomes are receiving increasing attention. Liver diseases such as hepatitis C virus (HCV) infection constitute an interesting paradigm for the biopsychosocial model. Before the availability of the new generation of antiviral drugs that directly inhibit HCV, treatment was largely based on interferon (IFN). However, IFN treatment gave the paradoxical effect of patients who were symptom-free but became ill because of the induced side effects of therapy. Among the most important adverse effects, depression (affecting up to 70% of patients) and common somatic symptoms (e.g., fatigue, insomnia, pruritus, diarrhea, flu-like syndrome, nausea, and headache) may lead to non-compliance and discontinuation of therapy (Schaefer et al., 2012). These drug-induced adverse effects are particularly challenging for clinicians due to their overlap with somatization symptoms. However, they are generally neglected by both physicians (because considered as expected side effects of therapy) and clinical psychologists (because considered as biological-driven symptoms), thereby perpetuating the organic-functional dualism (Sirri et al., 2013; Carrozzino et al., 2017; Fava et al., 2017). Evidence however showed that psychological constructs such as proneness to somatization and higher depressive symptoms may explain not only poorer psychosocial functioning, but also higher perception and the reporting of somatic side effects (Porcelli et al., 2014a; Cozzolongo et al., 2015).

Among the different psychological constructs extensively investigated in several medical settings, alexithymia has received increasingly high attention in the last decades (Nemiah, 1977; Sifneos, 1994). Alexithymia is a multifaceted personality construct that represents a deficit in the cognitive processing of emotions. It is currently conceived as composed by two higher order factors including deficit of affect awareness (difficulty identifying and describing feelings) and operatory thinking [externally oriented thinking (EOT) and poor imaginal processes; (Taylor et al., 1997; Bagby et al., 2006)]. These main characteristics of alexithymia reflect a defective cognitive processing of feelings that is thought to generate an individual inability to regulate affects and emotions (Taylor et al., 1997). Although alexithymia was early considered as one of the core personality determinants of psychosomatic illness (Nemiah and Sifneos, 1970; Nemiah et al., 1976), current evidence shows that the alexithymic deficit in processing feelings is an unspecific vulnerability factor and a trans-categorical dimension rather than a specific category. More specifically, alexithymia is likely to affect health in different ways as follows: (1) by influencing affective states, e.g., altered eating-related behavior; (2) through somatosensory amplification leading to low tolerance to painful stimuli (e.g., chronic pain); (3) through a post-traumatic shutdown of emotions (e.g., acute reactions to illness); (4) by altered autonomic, endocrine, and immune activity leading to tissue damage (e.g., increased vulnerability to inflammatory processes) (Kooiman et al., 2000; Lumley et al., 2007).

Alexithymia has been conceived as the inability to tolerate negative affect by balancing it with positive affect without mostly relying on external reality such as medical reassurance or behavioral actions such as drugs usage (Taylor et al., 1997; Taylor and Bagby, 2012). The concept of alexithymia as a personality construct of affect dysregulation is supported by separate lines of research. One is based on neuroimaging studies suggesting impairment in integration of interhemispheric transfer communication and dysregulation over prefrontal cortex and anterior regions (Moriguchi et al., 2006; Kano et al., 2007; Karlsson et al., 2008). The other is based on the high prevalence rate of alexithymia that has been found in a variety of chronic medical disorders, such as dermatology (Willemsen et al., 2008), cardiology (Tolmunen et al., 2010), pain disorders (Di Tella and Castelli, 2016), and cancer (De Vries et al., 2012), as well as in many psychiatric disorders. Examples are provided by the clinical link between alexithymia and somatization (De Gucht and Heiser, 2003), as well as by the relationship of alexithymia with eating disorders, substance use disorders, and panic disorder (Taylor et al., 1997; Taylor and Bagby, 2012). In sum, evidence suggests that alexithymia is a defective processing of feelings that is likely to affect mental and somatic health through behavioral patterns such as altering the normal regulation of affective states, reactive shutdown of emotions, increased vulnerability to inflammatory processes, somatosensory amplification, adoption of an abnormal illness behavior (Lumley et al., 2007).

The various methods assess the alexithymia construct under different perspectives, each one with merits and limitations. Differences among assessment instruments are related to the nature of the instrument (e.g., self-report scales, interview-based questionnaires, by-proxy assessment, performance-based measures) the evaluation of different facets of the construct (e.g., emotionality, fantasy, interpersonal relationships, and cognitive aspects), the amount of time needed for administration (some scales are brief and easy to use, while others are longer and more time-consuming) specific linkage to the construct (some methods assess core aspects of alexithymia, whereas others are more closely tied to the overall construct). The 20-item Toronto Alexithymia Scale (TAS-20) (Bagby et al., 1994) is the third in the family of TAS scales, after the 26-item version (Taylor et al., 1985) and the less used TAS-Revised (Taylor et al., 1992). The TAS-20 is surely the most popular and used scale for assessing alexithymia. The TAS-20 has been widely supported psychometrically in terms of good reliability and acceptable construct validity. In particular, its 3-factor solution for difficulty identifying feelings (DIF), difficulty describing feelings (DDF), and EOT has been identified as stable and invariant across languages and cultures (Bagby et al., 1988; Taylor, 2000; Parker et al., 2003; Taylor et al., 2003; Taylor and Bagby, 2004). The availability of a reliable, valid, short, not time-consuming, and easy-to-administer measure contributed substantially to the expansion of research on alexithymia over time. In other words, this common single instrument permitted the accumulation of a large body of knowledge, through the use of a single common instrument, as well as a large consensus in considering the TAS-20 as a “gold standard” for assessing the construct of alexithymia. The TAS-20 has been however criticized for not including items that directly assess the reduced fantasy and imaginal thinking facets, from one side, and for a sort of paradox because of its self-report nature. It is indeed highly controversial that individuals supposed to lack of introspective skills may be able to accurately rate their deficits in affective awareness (Lane et al., 2000; Kooiman et al., 2002).

Other assessment methods have been developed in response to the conceptual limitations of the TAS scales, even though basically the current knowledge on the multiple aspects of alexithymia rests on the TAS-26 and TAS-20 as the other measures have not reached the same level of usage as the Toronto scales. The Bermond-Vorst Alexithymia Questionnaire (BVAQ) (Vorst and Bermond, 2001) is a self-report scale, but the developers included items covering not only the cognitive dimension of alexithymia, but also its fantasizing and emotionalizing components. The Levels of Emotional Awareness Scale (LEAS) (Lane et al., 1990) is an indirect self-report measure as it requires to infer the presence of alexithymia by assessing trait emotional awareness. Other scales provide a direct assessment, but from a different perspective. For example, the Observer Alexithymia Scale (OAS) (Haviland et al., 2001) is a by-proxy measure that asks clinicians, family members, and acquaintances to rate the subject in order to overcome the self-report paradox. Other direct measures not based on self-reporting are structured and semi-structured interviews such as the Toronto Structured Interview for Alexithymia (TSIA) (Bagby et al., 2006) and the Diagnostic Criteria for Psychosomatic Research (DCPR) for alexithymia (Fava et al., 2017). Also, measures for assessing implicit motives such as Rorschach-based scores have been developed (Porcelli and Meyer, 2002; Tibon et al., 2005; Porcelli and Mihura, 2010). They are based on the assumption that this performance-based test is thought to infer the emotional ability (affective awareness and emotional expression) of an individual. However, these last scales have not been widely used because of their theoretical problems, practical difficulty in administration, insufficient statistics, large amount of time required for administration, and required extensive experience from the assessors. At this time, therefore, the substantial body of evidence on alexithymia is based on the TAS that is judged to be the best instrument for balancing sound characteristics and limitations.

To our knowledge no previous report systematically reviewed the body of literature investigating the role of alexithymia in gastroenterology. The present review aims to provide a synthesis of findings and to elucidate the clinical relevance of alexithymia in gastroenterology and hepatology. The inclusion of papers in this review was limited to those assessing alexithymia with the TAS as the use of this single instrument allows comparing prevalence and findings across different settings, population, and disorders.

Based on findings in other medical conditions (Lumley et al., 1996), we expected that alexithymia would be:

(1) more prevalent in functional than organic GI disorders because of the supposed higher impact of psychological factors in medical syndromes whose clinical outcomes are based more on the subjective perception of somatic health.

(2) more clinically relevant, according to Lipowski's view (1970), in illness dimensions related to somatization and/or somatoform-like symptoms.

## Methods

### Eligibility criteria

Eligible articles included only original research studies (e.g., brief research reports, short communications, research letters, research articles, single cases, meta-analytic studies, as well as other studies reporting quantitative data by focusing on a specific experimental design) that were published in peer-reviewed journals and were written in English. As a consequence, qualitative studies (e.g., reviews, commentaries, letters to the editor, books or book chapters) were excluded. Also, studies had:

1. to investigate a defined GI disorder. The most frequently investigated GI conditions are FGID as IBS, FD, functional abdominal pain syndrome, and other syndromes that are included in the panel of the Rome criteria (Drossman, 2016), and IBD as UC and CD;
2. to investigate a defined liver disease as HCV infection, cirrhosis, and liver transplantation (LT);
3. to use one of the three versions of the Toronto Alexithymia Scale, i.e., the TAS-26 (Taylor et al., 1985), the revised TAS (TAS-R) (Taylor et al., 1992), and the 20-item version of the TAS (TAS-20) (Taylor et al., 1992; Bagby et al., 1994) (see Introduction).

### Information sources and literature search

The International Preferred Reporting Items for Systematic Reviews and Meta-Analyses (PRISMA) guidelines (Moher et al., 2009) were used during the systematic search procedure of literature on PsycInfo, Medline, PubMed, Web of Science, Scopus, Cochrane, and ScienceDirect databases. Each database was systematically searched from inception (1985, i.e., when the TAS-26 was published) to July 2017. In order to further screen for relevant studies that may have been omitted by the systematic search, a manual search of the literature was also performed on Google Scholar. When screening the research literature for titles, abstracts, and keywords, specific search terms were used and combined in the following Boolean string: (“alexithymia” OR “alexithymic”) AND (“gastroenterology” OR “ulcerative colitis” OR “irritable bowel syndrome” OR “functional dyspepsia” OR “inflammatory bowel disease” OR “hepatology” OR “liver disease” OR “hepatitis C” OR “cirrhosis”).

### Selection of articles and data extraction

One of the authors (D.C.) performed the initial data extraction by removing duplicates and all the articles that appeared clearly irrelevant on the basis of the relevance of the title and after reading the specific abstract. The full-text of the remaining studies were independently assessed for eligibility by both authors. After a full-text evaluation of the potentially relevant studies, the two authors reached a consensus regarding eligibility and excluded all the research articles that not meet the inclusion criteria.

### Analysis of articles and data synthesis

In view of the highly heterogeneous clinical populations, including patients with different GI disorders and liver diseases, a meta-analysis was not deemed to be fully appropriate from a psychometric point of view. On this basis, for each of the included studies, we provided a qualitative synthesis of relevant data. More specifically, we analyzed data regarding objective of the study, number and description of participants by reporting the main results obtained with the TAS (e.g., mean scores, and prevalence of alexithymia).

## Results

### Included studies

**Figure 1:**
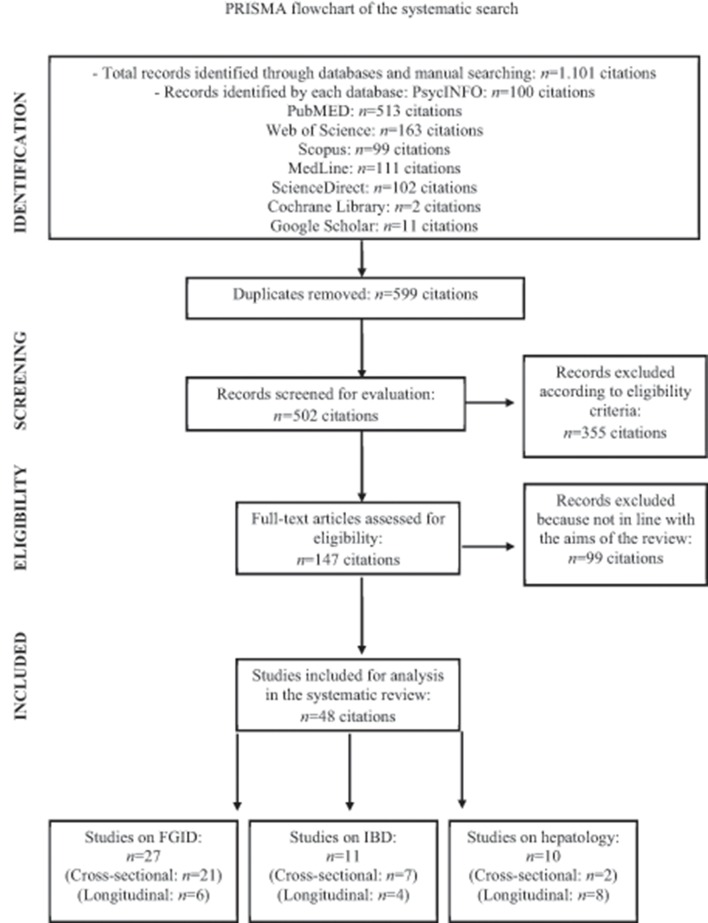
PRISMA flowchart of the systematic search.

As presented in the PRISMA flow chart (Figure 1), a total of 1,101 studies were identified by the systematic search. After removing 599 duplicates and excluding 355 clearly irrelevant studies, the full-text of the remaining 147 articles was evaluated for potential eligibility. Of these studies, 99 articles were further discarded because of the following: (1) alexithymia was not measured with one of the three versions of the TAS; (2) the clinical samples did not include patients with GI disorders or liver diseases; (3) the manuscript was not written in English; (4) the study consisted of a conference paper and only an abstract without full-text was available; (5) the study did not have a quantitative experimental design. Of the remaining 48 full-text articles that were found eligible for inclusion, no unpublished studies were reported. Overall, 27 studies included patients with FGID, of which 21 cross-sectional and 6 longitudinal studies; 11 IBD patients, of which 7 cross-sectional and 4 longitudinal studies; and 10 patients with liver diseases, of which 2 cross-sectional and 8 longitudinal studies. Tables 1–6 showed the characteristics of the included reports.

**Table 1:** Cross-sectional studies on alexithymia in functional gastrointestinal disorders.

**Authors**	**Aim**	**Clinical sample**	**Control sample**	**Mean (SD) scores on TAS**	**Prevalence (%) of alexithymia**	**Main results**
Arun, 1998	Association of alexithymia with neuroticism, depression, and stressful life events	30 consecutive IBS patients	30 healthy individuals	IBS patients: 125.96 ± 11.5^**^ Control sample: 108.96 ± 13.29	–	Compared to subjects with low alexithymia (3.65 ± 2.52), those with high alexithymia reported significantly higher neuroticism scores (8.67 ± 4.75) (*F ratio* = 6.52; *p* < 0.01)
Bengtsson et al., 2013	Comparison of psychological and coping functioning between IBS and IBD patients	81 patients with IBS 74 patients with IBD	None	IBS patients: median (interquartile ranges) = 44 (36-54)^*^ IBD patients: median (interquartile ranges) = 42 (34-50)	–	IBS patients scored higher on the TAS-20 than those with IBD, but no statistically significant difference was found when adjusted for gender and age (*OR* = 1.025; 95% *CI* = 0.993–1.057; *p* = 0.123)
Dibaise et al., 2016	Evaluation of the relationship between psychological dysfunction, gastric emptying, and dyspeptic symptoms	Out of 209 patients undergoing gastric emptying testing, 151 met the Rome III criteria for FD	None	FD patients: 47.62 ± 13.06^*^ Non-FD patients: 45.62 ± 13.32	–	There were no statistically significant differences on the TAS-20 between FD and non-FD patients (*Cohen's d* = 0.14; *p* = 0.332)
Eiroa-Orosa et al., 2015	Evaluation of psychological, subjective functionality	28 FGID patients and 17 patients with gastrointestinal motility disorders	None	FGID patients: 46.16 ± 13.91^*^ Patients with gastrointestinal motility disorders: 48.43 ± 17.15	–	FGID patients were not more alexithymic than those with GI motility disorders (*z* = −0.63; *p* = 0.527)
Faramarzi et al., 2012	Association between psychological symptoms, including alexithymia, and the occurrence of FD	60 consecutive FD patients	60 healthy controls	FD patients: 61.58 ± 6.56^*^ Healthy controls: 47.50 ± 8.30	–	Compared to healthy subjects, FD patients reported significantly higher alexithymia (*F* = 106.16; *p* < 0.001)
Faramarzi et al., 2014	Evaluation of psychiatric problems, including alexithymia	30 FD patients 30 PUD patients	30 healthy individuals	FD patients: 62.9 ± 4.7^*^ PUD patients: 62.2 ± 10.6 Healthy controls: 55.6 ± 10.4	–	FD patients reported significantly more alexithymia than those with PUD (62.9 ± 4.7 vs. 62.2 ± 10.6; *adj R*^2^ = 0.10; *F* = 5.96; *p* = 0.004)
Farinelli et al., 2007	Prevalence of alexithymia in GERD	69 patients with GERD	None	43.7 ± 9.9^*^	0%	GERD patients were not alexithymic according to TAS-20 cut-off scores
Jones et al., 2004	Evaluation of alexithymia and somatosensory amplification	111 patients with FD	53 healthy subjects	FD patients: 43 ± 11^*^ Healthy subjects: 38 ± 9	FD patients: 12% Healthy controls: –	FD patients scored significantly higher than controls on TAS-20 (*t*-test; *p* < 0.005) Difficulty in identifying feelings significantly correlated with abdominal discomfort (*r* = 0.27) and bloating (*r* = 0.23)
Jones et al., 2006	Evaluation of coping strategies, social support and psychological factors, including alexithymia	74 IBS patients 48 IBD patients	55 healthy subjects	IBS patients: 42 ± 12^*^ IBD patients: 43 ± 12 Controls: 38 ± 9	IBS patients: 8% IBD patients: 8% Controls: 0%	Alexithymia was detected in 6 patients with IBS, in 4 IBD patients, and in 0 controls (χ^2^ = 4.78; *p* = 0.09)
Jones et al., 2013	Association between abuse history, state affect, somatic symptom reporting, trait affectivity, including alexithymia, gastric discomfort threshold and health-related quality of life	259 consecutive FD patients	None	50.9 ± 12.8^*^	–	Higher levels of alexithymia and trait anxiety were significantly associated with lower mental quality of life (*effect size* = −1.12; *factor loadings/standardized path coefficients* = 0.23; *p* < 0.001)
Mazaheri et al., 2012	Evaluation of alexithymia and severity of GI symptoms	129 FGID patients	109 healthy individuals	FGID patients: 60.26 ± 11.18^*^ Control group: 21.62 ± 3.2	–	FGID patients scored significantly higher on TAS-20 than controls (*F* = 1.20; *df* = 1; *p* < 0.001)
Phillips et al., 2013	Evaluation of psychosocial factors, including alexithymia	82 patients with IBS	67 healthy controls	IBS patients: 50.8 ± 12.5^*^ Healthy controls: 43.4 ± 12.2	–	Alexithymia factors as difficulty identifying feelings, and difficulty describing feelings were two significant predictors of IBS symptom severity
Porcelli et al., 1999	Evaluation of alexithymia	121 consecutive FGID outpatients 116 IBD outpatients	112 healthy individuals	FGID patients: 62.7 ± 13.6^*^ IBD patients: 54.3 ± 13.9 Healthy controls: 42.9 ± 9.1	FGID patients: 66% IBD patients: 38% Healthy subjects: 4.5%	FGID patients were significantly more alexithymic than IBD and control groups [*F*_(2, 347)_ = 73.30; *p* = 0.001]
Porcelli and De Carne, 2001	Validation of the DCPR diagnostic criteria for alexithymia	190 consecutive FGID outpatients	None	59.6 ± 13.9^*^	60%	FGID patients meeting the DCPR criteria for alexithymia scored significantly higher on the TAS-20 than those who were negatives (*t* = 9.86; *p* < 0.001)
Porcelli et al., 2004	Evaluation of alexithymia and psychopathology	58 FGID patients of which 38 with psychiatric comorbidities 52 psychiatric patients of which 25 with comorbid FGID	None	FGID patients: 64.6 ± 12.4^*^ Psychiatric patients: 50.8 ± 12.9 FGID patients with psychiatric comorbidities: 66.7 ± 11.2 Psychiatric patients with FGID comorbidities: 52.7 ± 10.4	FGID patients: 75.9% Psychiatric patients: 21.1% FGID patients with psychiatric disorders: 81.6% Psychiatric patients with FGID: 28%	Alexithymia independently predicted which subjects belonged to the subgroup of FGID patients with psychiatric comorbidities (*OR* = 1.14; 95% CI = 1.03–1.26; *p* = 0.001)
Porcelli et al., 2014b	Influence of alexithymia and GSA on IBS severity	177 outpatients with IBS	None	65.33 ± 7.22^*^	54.2%	Alexithymia explained more unique variance than GSA in predicting IBS symptom severity.
van Kerkhoven et al., 2006	Influence of alexithymia on GI endoscopy outcomes	1141 consecutive FGID patients	None	51 ± 12^*^	21%	Patients with alexithymia reported more GI symptoms (6 ± 4 vs. 5 ± 3; *p* < 0.05) and higher symptom severity (42 ± 34 vs. 34 ± 30; *p* < 0.01) than those who were not alexithymic
Van Oudenhove et al., 2008	Influence of psychosocial factors, including alexithymia, on gastric symptom severity	201 consecutive FD patients	None	Median (interquartile range): 53 (42-61)^*^	87%	Out of 201 FD patients, 174 were alexithymic on the basis of the TAS-20 cut-off score
Van Oundenhove et al., 2011a	The role of gastric sensorimotor function, psychological and psychiatric comorbidities, including alexithymia, on comorbid IBS and CF-like symptoms	259 consecutive FD patients of which 70 without comorbidity, 80 with comorbid IBS, 40 with comorbid fatigue, and 61 with comorbid IBS and fatigue	None	FD patients without comorbidity: 15.5 ± 6.0^*^ FD patients with comorbid IBS: 16.4 ± 6.1 FD patients with comorbid CF-like symptoms: 17.7 ± 6.6 FD patients with comorbid IBS and CF-like symptoms: 17.4 ± 6.5	–	When comparing groups according to comorbidity (none, IBS alone, fatigue alone, or both comorbidities), no statistically significant difference was found on the TAS-20 (*one-way ANOVA*; *p* = 0.25)
Van Oudenhove et al., 2011b	Influence of gastric sensorimotor function, psychosocial factors, including alexithymia, and somatization on quality of life	259 consecutive FD patients	None	–	–	The mental component of health-related quality of life was negatively and significantly associated with alexithymia (Spearman's *rho* = −0.42)
Weinryb et al., 2003	Evaluation of psychological factors, including alexithymia	17 IBS patients 17 IBS non patients	17 healthy controls	IBS patients: 42.3 ± 14.1^*^ IBS non patients: 39.6 ± 11.4 Controls: 32.0 ± 8.8	–	Patients with IBS and controls were not-alexithymic and there were no statistically significant differences between groups on the TAS-20 (*F* = 3.56; *p* = 0.044)

*CF, chronic fatigue; DCPR, Diagnostic Criteria for Psychosomatic Research; FD, functional dyspepsia; FGID, functional gastrointestinal disorders, including functional dyspepsia, irritable bowel syndrome, functional abdominal pain syndrome; GERD, Gastroesophageal Reflux Disease; GI, gastrointestinal; GSA, gastrointestinal-specific anxiety; IBD, inflammatory bowel disease, including patients diagnosed with ulcerative colitis or with Crohn's disease; IBS, irritable bowel syndrome; PUD, Peptic Ulcer Disease; TAS, Toronto Alexithymia Scale*.

* *Using TAS-20 for evaluating alexithymia*.

** *Using TAS-26 for evaluating alexithymia*.

**Table 2:** Longitudinal studies on alexithymia in functional gastrointestinal disorders.

**Authors**	**Aim**	**Clinical sample**	**Control sample**	**Mean (SD) scores on TAS**	**Prevalence (%) of alexithymia**	**Main results**
Faramarzi et al., 2013	Influence of brief core conflictual relationship theme psychoanalytic psychotherapy on GI symptoms and alexithymia	Out of 49 patients with FD, 24 were randomly assigned to the experimental group	Out of 49 patients with FD, 25 were randomly assigned to the control group	FD patients assigned to the experimental group at baseline: 63.5 ± 5.2^*^ FD patients assigned to the experimental group after treatment: 47.2 ± 9.0 FD patients assigned to the experimental group at follow-up 1: 47.9 ± 10.0 FD patients assigned to the experimental group at follow-up 2: 47.7 ± 9.8 FD patients assigned to the control group at baseline: 60.4 ± 8.1 FD patients assigned to the control group after treatment: 60.9 ± 8.9 FD patients assigned to the control group at follow-up 1: 57.0 ± 10.4 FD patients assigned to the control group at follow-up 2: 60.3 ± 8.5	–	Brief psychodynamic psychotherapy significantly improved all GI symptoms, as well as many psychological symptoms as alexithymia
Farnam et al., 2014	Influence of emotional awareness training on the severity and frequency of pain and alexithymia	100 IBS patients	587 university students	IBS patients: 59.67 ± 9.92^*^ Healthy controls: 43.88 ± 15.06	–	Patients with IBS scored significantly higher than controls on the TAS-20 (*t*-test; *p* < 0.005)
Porcelli et al., 2003	Influence of alexithymia on treatment outcomes	At baseline: 130 consecutive FGID patients After 6 months of follow-up: 112 FGID patients	None	Baseline: 58.89 ± 13.50^*^ Follow-up: 55.85 ± 12.32	56%	Even after controlling for baseline anxiety and depression, alexithymia emerged as the most powerful predictor of both recovery status and improvement of GI symptoms
Porcelli et al., 2007a	Role of alexithymia on the persistence of gastrointestinal symptoms	Out of 52 gallstone patients judged as also having dyspepsia, 31 patients were categorized as improved and 21 as unimproved	None	Improved patients: 49.32 ± 11.50^*^ Unimproved patients: 66.00 ± 7.79	–	Alexithymia significantly predicted the persistence of GI symptoms more strongly than did psychological distress
Porcelli et al., 2017	Influence of alexithymia and GSA on symptom improvement	150 patients with IBS	None	Improved IBS patients at baseline: 55.97 ± 12.22^*^ Improved IBS patients at follow-up: 51.49 ± 10.18 Unimproved IBS patients at baseline: 63.46 ± 7.35 Unimproved IBS patients at follow-up: 63.92 ± 8.54	Improved IBS patients at baseline: 45.9% Improved IBS patients at follow-up: 24.3% Unimproved IBS patients at baseline: 84.6% Unimproved IBS patients at follow-up: 82%	Alexithymia, but not GSA, significantly predicted post-treatment improvement of GI symptoms
Portincasa et al., 2003	Evaluation of gastrointestinal motility and alexithymia	100 consecutive patients with IBS	100 healthy participants	IBS patients: 59.1 ± 1.1^*^ Healthy controls: 40.5 ± 1.0	IBS patients: 43% Healthy controls: 2%	Alexithymia positively and significantly correlated with dyspepsia symptoms (*r* = 0.47) and frequency of bowel movements (*r* = 0.52)

*FD, functional dyspepsia; FGID, functional gastrointestinal disorders, including functional dyspepsia, irritable bowel syndrome, functional abdominal pain syndrome; GI, gastrointestinal; GSA, gastrointestinal-specific anxiety; IBS, irritable bowel syndrome; TAS, Toronto Alexithymia Scale*.

* Using TAS-20 for evaluating alexithymia. ^*^^*^Using TAS-26 for evaluating alexithymia.

**Table 3:** Cross-sectional studies on alexithymia in inflammatory bowel disorders.

**Authors**	**Aim**	**Clinical sample**	**Control sample**	**Mean (SD) scores on TAS**	**Prevalence (%) of alexithymia**	**Main results**
Boye et al., 2008a	Influence of some personality factors, including alexithymia, on quality of life of IBD patients	110 IBD patients, of which 56 with UC, and 54 with CD	None	UC patients: 65.5 ± 10.7^**^ CD patients: 67.0 ± 10.1	–	In patients with UC, alexithymia significantly and negatively correlated with the emotional domain of quality of life (*r* = −0.26)
Boye et al., 2008b	Influence of some personality traits, including, alexithymia on general quality of life	109 IBD patients	None	CD patients: 67.0 ± 10.1^**^ UC patients: 65.5 ± 10.7	–	In patients with UC, high levels of alexithymia were significantly associated with low levels in the physical (*r* = −0.33), mental (*r* = −0.49), and social (*r* = −0.30) components of health-related quality of life In CD patients, high levels of alexithymia significantly correlated with increased physical limitations (*r* = 0.29)
Iglesias-Rey et al., 2012	Influence of alexithymia on health-related quality of life	484 IBD patients of which 192 with CD, and 292 with UC	None	Total sample (95% CI): 65.8 (95% CI = 64.6–67.0) ^**^ CD patients: 65.2 (95% CI = 63.1–67.3) UC patients: 66.2 (95% CI = 64.7–67.6)	Total IBD sample: 30.2% CD patients: 30.5% UC patients: 30.0%	Multivariate logistic regression analysis found that alexithymia was a risk factor for poor quality of life (*OR* = 3.34; 95% CI = 1.98–5.65)
La Barbera et al., 2017	Association between IBD and specific psychological factors, including alexithymia	100 IBD patients	66 healthy individuals	R1 community containing 15 CD and 24 UC patients: 40.0 ± 10.3^*^ R1 community containing 47 control participants: 43.2 ± 8.4 R2 community containing 40 CD and 21 UC patients: 53.1 ± 12.3 R2 community containing 19 control participants: 53.5 ± 15.0	–	It has been found a significant correlation between alexithymia and low levels of physical and mental health
Porcelli et al., 1995	Association between alexithymia and IBD	112 IBD patients, of which 89 with UC, and 23 with CD	112 healthy subjects	IBD patients: 53.7 ± 13.6^*^ UC patients: 53.1 ± 13.0 CD patients: 55.5 ± 15.9 Healthy controls: 42.8 ± 8.9	IBD patients: 35.7% Healthy controls: 4.5%	Patients with IBD scored significantly higher than controls on the TAS-20 (*t* = 7.02; *p* < 0.001)
Verissimo et al., 1998	The influence of alexithymia on health-related quality of life	74 IBD patients, of which 49 with CD, and 25 with UC	None	Total sample: 51.2 ± 9.7^*^ CD patients: 51.5 ± 9.3 UC patients: 50.8 ± 10.8	–	Alexithymia correlated negatively and significantly with quality of life (*r* = −0.34), bowel symptoms (*r* = −0.28), systemic symptoms (*r* = c0.37), and emotional functioning (*r* = −0.38)
Verissimo et al., 2000	Relationship between alexithymia and locus of control	124 patients, of which 74 with IBD	218 medical students	Patients: 56.44 ± 11.66^*^ Controls: 48.68 ± 9.15	–	Patients scored significantly higher than controls on the TAS-20 total scale (*t* = 6.82; *p* < 0.001)

*CD, Crohn's Disease; IBD, inflammatory bowel disease, including patients diagnosed with ulcerative colitis or with Crohn's disease; TAS, Toronto Alexithymia Scale; UC, Ulcerative Colitis*.

* *Using TAS-20 for evaluating alexithymia*.

** *Using TAS-26 for evaluating alexithymia*.

**Table 4:** Longitudinal studies on alexithymia in inflammatory bowel disorders.

**Authors**	**Aim**	**Clinical sample**	**Control sample**	**Mean (SD) scores on TAS**	**Prevalence (%) of alexithymia**	**Main results**
Porcelli et al., 1996	Influence of IBD activity on both alexithymia and psychological distress	104 patients with IBD	None	Unchanged IBD patients at baseline: 53.9 ± 13.4^*^ Improved IBD patients at baseline: 55.0 ± 13.9 Worsened IBD patients at baseline: 53.1 ± 14.6	–	TAS-20 scores at baseline significantly and impressively correlated with TAS-20 scores at follow-up (*r* = 0.95) A series of ANOVA measures revealed also that there was no influence of disease activity on alexithymia levels
Porcelli and Meyer, 2002	Evaluation of the construct validity of Rorschach alexithymia variables	92 outpatients with IBD were divided into the following three groups: (1) 32 IBD patients with stable alexithymia; (2) 45 IBD patients without alexithymia; (3) 15 IBD patients with indeterminate alexithymia	None	IBD patients with alexithymia at baseline: 66.4 ± 4.5^*^ IBD patients with alexithymia at follow-up: 66.6 ± 3.2 Not-alexithymic IBD patients at baseline: 41.0 ± 5.5 Not-alexithymic IBD patients at follow-up: 41.2 ± 4.3 IBD patients with indeterminate alexithymia at baseline: 53.5 ± 6.1 IBD patients with indeterminate alexithymia at follow-up: 53.3 ± 7.0	34.8% of IBD patients presented with stable alexithymia 48.9% of IBD patients presented without alexithymia 16.3% of IBD patients presented with indeterminate alexithymia	Compared to IBD patients with indeterminate alexithymia or without alexithymia, those presenting with stable alexithymia showed an impoverished fantasy life, poorly adapted emotional expression, poor coping resources, concrete and stereotypical thinking, as well as social conformity with compromised relationships
Porcelli and Mihura, 2010	Evaluation of the alexithymia Rorschach scale	92 patients with IBD 127 psychiatric outpatients	None	IBD patients: 52.1 ± 12.1^*^ Psychiatric outpatients: 52.2 ± 12.9	Out of 219 patients, 38.8% (*n* = 85) were alexithymic	IBD patients and those with psychiatric disorders had similar levels of alexithymia
Tibon et al., 2005	Evaluation of the construct validity of a new Rorschach index,	92 IBD patients	None	–	34.8%	Compared to IBD patients with indeterminate alexithymia on the TAS-20, those with alexithymia scored significantly higher on the Rorschach Reality-Fantasy Scale

*IBD, inflammatory bowel disease, including patients diagnosed with ulcerative colitis or with Crohn's disease; TAS, Toronto Alexithymia Scales*.

* *Using TAS-20 for evaluating alexithymia*.

** Using TAS-26 for evaluating alexithymia.

**Table 5:** Cross-sectional studies on alexithymia in hepatology.

**Authors**	**Aim**	**Clinical sample**	**Control sample**	**Mean (SD) scores on TAS**	**Prevalence (%) of alexithymia**	**Main results**
Blomhoff and Malt, 1995	Behavioral styles in non-alcoholic liver transplant candidates	29 patients with advanced liver disease	None	Patients: 64.0 ± 10.5^**^	–	No one of patients were alexithymic on the basis of TAS-26 mean scores
Rustgi et al., 2010	The association of alexithymia with a variety of GI symptoms	83 consecutive HCV outpatients	None	48.01 ± 12.82^*^	22%	Compared to not alexithymic patients, those with alexithymia had greater viral loads and reported more subjective appraisal for their illness

*GI, gastrointestinal; HCV, Hepatitis C; TAS, Toronto Alexithymia Scale*.

* *Using TAS-20 for evaluating alexithymia*.

** *Using TAS-26 for evaluating alexithymia*.

**Table 6:** Longitudinal studies on alexithymia in hepatology.

**Authors**	**Aim**	**Clinical sample**	**Control sample**	**Mean (SD) scores on TAS**	**Prevalence (%) of alexithymia**	**Main results**
Cozzolongo et al., 2015	Role of alexithymia in the quality of life of patients with chronic HCV treated with antiviral therapy	124 HCV patients	None	Male HCV patients: 53.63 ± 13.36^*^ Female HCV patients: 46.71 ± 10.14	33.1%	Poor quality of life was significantly and independently predicted by alexithymia and psychological distress before (*R*^2^ = 0.60) and 6 months after the antiviral treatment (*R*^2^ = 0.69)
Fukunishi et al., 2002	Evaluation of post-transplant occurrence of psychiatric disorders, including alexithymia	31 recipient-donor pairs who underwent living-related liver transplantation 65 recipient-donor pairs who underwent living-related kidney transplantation	None	–	Pre-living-related liver transplantation recipients: 38.7% Pre-living-related liver transplantation donors: 9.7% Pre-living-related kidney transplantation recipients: 41.5% Pre-living-related kidney transplantation donors: 12.3%	Compared to not-alexithymic recipients, those with alexithymia undergoing a liver (χ^2^ = 7.2; *df* = 1; *p* = 0.007) or a kidney transplantation (χ^2^ = 19.7; *df* = 1; *p* < 0.001) exhibited significantly more paradoxical psychiatric syndrome
Fukunishi et al., 2003	Evaluation of alexithymia before and after transplantation	116 pairs of recipients and donors who underwent living-related liver transplantation 325 pairs of recipients and donors who underwent living-related kidney transplantation	None	–	–	In recipients undergoing a liver or a kidney transplantation, the severity of alexithymia was significantly higher before than after transplantation
Murri et al., 2017	The role of different biopsychosocial risk factors, including alexithymia, on the onset of depression	55 HCV patients of which 29 classified as depressed, and the remaining 26 as not depressed	None	Depressed HCV patients: 52.3 ± 16.4^*^ Not depressed HCV patients: 48.5 ± 13.9	–	HCV patients developing interferon-related persistent depression had higher TAS-20 scores, but this difference did not reach statistical significance (*t* = 1.62; *p* = 0.11)
Nardelli et al., 2013	Influence of depression, anxiety and alexithymia on health related quality of life	60 cirrhotic patients	None	–	23%	All domains of health-related quality of life, but bodily pain, were significantly impaired in patients with alexithymia
Palmieri et al. (2013)	Evaluation of psychopathological factors, including alexithymia	22 cirrhotic patients 24 patients with hepatocellular carcinoma	20 healthy controls	Cirrhotic patients: 59.6 ± 16.2^*^ Patients with hepatocellular carcinoma: 60.8 ± 15.1 Healthy controls: 36.5 ± 8.9	Cirrhotic patients: 54% Patients with hepatocellular carcinoma: 50%	Both cirrhotic patients and those with hepatocellular carcinoma were significantly more alexithymic than controls
Porcelli et al., 2014a	Influence of somatization and alexithymia on somatic symptom reporting	111 chronic HCV patients	None	50.72 ± 12.37^*^	–	Alexithymia and somatization significantly and independently predicted somatic adverse events (as fatigue, pain, and skin, digestive, respiratory, and ophthalmological disturbances) both at baseline and follow-up
Porcelli et al., 2015	Role of alexithymia and specific functional gene variants on depression	130 consecutive patients presenting with chronic HCV	None	HCV patients with HTR1A-G/G: 62.08 ± 9.37^*^ HCV patients with 5-HTTLPR-s/s: 48.23 ± 11.45	HCV patients with HTR1A-G/G: 20% HCV patients with 5-HTTLPR-s/s: 17.7%	Alexithymia and the G-variant polymorphism of the HTR1A (a promoter region of the serotonin receptor gene), both separately and jointly, significantly predicted the development of interferon-induced depression

*HCV, Hepatitis C; TAS, Toronto Alexithymia Scale*.

* *Using TAS-20 for evaluating alexithymia*.

** Using TAS-26 for evaluating alexithymia.

### FGID and alexithymia

FGID are a multifactorial group of disorders of the GI tract whose pathogenesis remains incompletely understood from a medical point of view (Koloski et al., 2012; Keightley et al., 2015). The etiology of these disorders may result from an altered communication of the bidirectional gut-brain axis that is not fully explained by medically known structural, biochemical or organic abnormalities (Van Oudenhove et al., 2016). From a biopsychosocial perspective, FGID may be considered a consequence of a complex dysregulation of brain-gut and affective systems, involving emotional, cognitive, and neurophysiological functions (Drossman, 2016). More specifically, motility disturbances of the GI tract and alterations in sensory input from the gut have been implicated and, by way of the bidirectional brain-gut axis, functional somatic symptoms may be generated by states of emotional arousal and by mutual interactions of the enteric, nervous, and immune systems (Mayer, 2011).

#### Prevalence of alexithymia in FGID

Most studies on FGID had a cross-sectional design (Arun, 1998; Porcelli et al., 1999; Porcelli and De Carne, 2001; Weinryb et al., 2003; Van Oudenhove et al., 2008; Van Oundenhove et al., 2011a; Faramarzi et al., 2012; Mazaheri et al., 2012; Bengtsson et al., 2013) and control groups were formed of healthy subjects (Arun, 1998; Porcelli et al., 1999; Faramarzi et al., 2012; Mazaheri et al., 2012). The first study investigating alexithymia with the original TAS-26 (Taylor et al., 1985) in a sample of 30 IBS patients showed they had higher levels of alexithymia (125.96 ± 11.5) than a control group of 30 matched healthy subjects (108.96 ± 13.29) (*t* = 20.04; *p* < 0.01) (Arun, 1998). In the same period, a similar study compared 121 consecutive patients with FGID with a sample of 116 IBD outpatients and a control group of 112 healthy participants (Porcelli et al., 1999). They found that FGID patients scored significantly higher on the TAS-20 (62.7 ± 13.6) than IBD respondents (54.3 ± 13.9) and healthy subjects (42.9 ± 9.1) [*F*_(2, 347)_ = 73.30; *p* = 0.001] FGID patients scored significantly higher (62.7 ± 13.6) than IBD patients (52.6 ± 12.5) on the TAS-20 [*t*_(162)_ = 4.21; *p* < 0.001], even after controlling for education, gender, anxiety, depression, and overall GI symptoms. When considered categorically by using the suggested TAS-20 cut-off scores (Taylor et al., 1997), the prevalence of alexithymia was 66% (*n* = 80) in the FGID, 38% (*n* = 44) in the IBD, and 4.5% (*n* = 5) in the control groups (χ^2^ = 94.94, *p* < 0.001). Similarly, it was found that 60 FD patients scored significantly higher than 60 controls on the total (61.58 ± 6.56 vs. 47.50 ± 8.30) as well as the DIF (27.45 ± 4.31 vs. 17.52 ± 4.47) and DDF (17.22 ± 4.10 vs. 13.00 ± 3.74) subscales, even after multiple adjustments (*F* = 106.16; *p* < 0.001) (Faramarzi et al., 2012). By contrast, there was no statistically significant difference between groups on the EOT factor of the TAS (16.92 ± 3.51 vs. 16.98 ± 3.31; *t*-test; *p* = 0.915).

Consistently, a sample of 100 IBS patients (Farnam et al., 2014) scored significantly higher than 587 healthy controls on the TAS-20 total score (59.67 ± 9.92 vs. 43.88 ± 15.06), as well as on all the three subscales (DIF: 18.60 ± 5.90 vs. 16.54 ± 6.55, DDF: 14.66 ± 4.25 vs. 11.44 ± 3.36, EOT:26.41 ± 3.09 vs. 15.88 ± 5.27). Similar findings were reported by Faramarzi et al. (2014) showing that TAS-20 total scale (62.9 ± 4.7 vs. 62.2 ± 10.6; *adj*.*R*^2^ = 0.10; *F* = 5.96; *p* = 0.004), as well as DIF (27.8 ± 3.9 vs. 23.5 ± 6.3; *adj.R*^2^ = 0.25; *F* = 16.07; *p* < 0.001), and DDF (17.3 ± 3.6 vs. 16.5 ± 4.4; *adj.R*^2^ = 0.58; *F* = 3.74; *p* < 0.028) scores were significantly higher in a group of 30 FD patients than 30 patients with peptic ulcer. These findings were consistent with another study evidencing high prevalence rates of alexithymia at 60% (*n* = 114) in a large sample of 190 FGID outpatients as measured by the TAS-20 and at 47.4% (*n* = 90) as identified by the DCPR alexithymia cluster (Porcelli and De Carne, 2001). These patients also scored significantly higher on the TAS-20 (65.2 ± 10.9) than patients not meeting the DCPR criteria for alexithymia (48.2 ± 11.4) (*t* = 9.86; *p* < 0.001). Similar results were obtained by Portincasa et al. (2003) on 100 patients with IBS who scored significantly higher on the TAS-20 (59.1 ± 1.1) than 100 healthy control subjects (40.5 ± 1.0). Similarly, they found higher prevalence of alexithymia in IBS patients (43%; *n* = 43) than healthy subjects (only 2%; *n* = 2). In this study, frequency of bowel movements was associated less with anxiety (*r* = 0.39; *p* < 0.001) and somatization (*r* = 0.34; *p* < 0.001) and more with alexithymia (*r* = 0.52; *p* < 0.001).

Similar findings of higher prevalence of alexithymia in FGID patients have been shown in other (as in Van Oudenhove et al., 2008; Mazaheri et al., 2012) but not all studies. In one small sample size study, no significant TAS-20 difference was found between 17 IBS (42.3 ± 14.1) and 17 healthy control groups (32.0 ± 8.8) (*F* = 3.56; *p* = 0.044; *effect size* = −0.88) (Weinryb et al., 2003). Also Bengtsson et al. (2013) found no statistical differences in the TAS-20 scores between 81 IBS and 74 IBD patients. Specifically, the TAS-20 scores were higher in the IBS sample, but no statistically significant difference was found when adjusted for gender and age (*OR* = 1.025; 95% CI = 0.993–1.057; *p* = 0.123). Similarly, no statistical difference was found by comparing TAS-20 scores of 70 FD patients without comorbidity (15.5 ± 6.0), 80 FD patients with comorbid IBS only (16.4 ± 6.1), 40 FGID patients with comorbid chronic fatigue-like symptoms only (17.7 ± 6.6), and 61 FD patients with comorbid IBS and fatigue (17.4 ± 6.5) (Van Oundenhove et al., 2011a).

#### Association with treatment outcome and symptom severity

Only few studies investigating the effects of alexithymia on treatment outcomes and symptom severity with a longitudinal design (Porcelli et al., 2003, 2007a, 2017). All the other studies included in this review (Porcelli et al., 2004, 2014b; Jones et al., 2006, 2013; van Kerkhoven et al., 2006; Farinelli et al., 2007; Van Oudenhove et al., 2011b; Phillips et al., 2013; Eiroa-Orosa et al., 2015; Dibaise et al., 2016) were cross-sectional. Moreover, all but two studies (Jones et al., 2006; Phillips et al., 2013) did not include control samples.

Porcelli et al. (2003) evaluated the stability of alexithymia by analyzing to what extent alexithymia can predict treatment outcomes in 112 patients with FGID after 6 months of as-usual care (various combination of psychological and pharmacological interventions). They showed that unimproved patients (*n* = 44) obtained significantly higher baseline TAS-20 scores (69.25 ± 7.81) than 68 improved patients (52.19 ± 12.10) (*t* = 7.89; *p* < 0.001). However the relative stability of alexithymia (i.e., the degree to which the relative differences of alexithymia among individuals remain the same over time, measured with Pearson test-retest coefficients) was established by correlating baseline and follow-up TAS-20 scores (*r* = 0.76, *p* < 0.001). When performing hierarchical regression models by analyzing the entire FGID sample, the authors were also able to show that baseline TAS-20 scores were significant predictors of follow-up TAS-20 scores even after controlling for baseline and follow-up depression (*R*^2^_*chg*_ = 0.23, *p* < 0.001) and anxiety (*R*^2^_*chg*_ = 0.35, *p* < 0.001) scores. Such findings suggest that the stability of TAS-20 scores over the 6 month treatment period could not be accounted for by their associations with anxiety and depression scores. To further determine the contribution of alexithymia as stable clinical factor in predicting treatment outcomes, a series of logistic and linear regression analyses showed that TAS-20 was the strongest predictor of treatment outcome, after controlling for co-variables, with an accurate prediction rate of 85% for the improved and 82% for unimproved patients. When alexithymia was entered into the regression model at a second step, after including anxiety, depression, and gastrointestinal symptoms at the step one, the overall fit of this model increased significantly, i.e., $\chi_{\text{dif}\left(1\right)}^{2}$ = 32.51, *p* < 0.001. The addition of alexithymia increased also the Cox and Snell *R*^2^ from 0.23 to 0.42.

GI-specific anxiety (GSA) (Labus et al., 2004, 2007) is another psychological factor that has been found to influence FGID. GSA refers to the cognitive, affective, and behavioral response stemming from fear of GI sensations or symptoms, and the context in which these visceral sensations and symptoms occur (Jerndal et al., 2010). GSA indeed relates to hypervigilance to, and fear, worry, and avoidance of GI-related sensations. Alexithymia and GSA are likely involved in visceral symptom perception since these psychological factors may indicate difficulty in emotional regulation, biased selective attention to somatic attention, higher negative emotionality, exaggerated symptom reporting, poor coping, avoidant behaviors, higher health anxiety resulting in heightened fear of GI symptoms (GSA) and difficulty identifying and describing feelings (alexithymia) (Labus et al., 2004, 2007). Consistent with these hypotheses, Porcelli et al. (2014b) found in 177 IBS patients that symptom severity was significantly and independently predicted by alexithymia and GSA at a similar degree, either when GSA was forced into the regression model before alexithymia (*R*^2^ = 0.51; Δ*R*^2^ = 0.14; β = 0.38), and alexithymia before GSA (*R*^2^ = 0.56; Δ*R*^2^ = 0.19; β = 0.53). Large effect sizes were found between symptom severity and alexithymia alone (*d* = 1.16) or combined with GSA (*d* = 1.45). However, TAS-20 alone explained 54% of the IBS severity score variance, to which GSA added a smaller although significant amount of 9%. In other words, alexithymia explained much more unique variance in IBS severity when compared with the contribution provided by GSA. Semipartial correlations that measure the proportion of IBS severity associated uniquely with the predictor confirmed that TAS-20 was associated more strongly with illness severity (*semipartial r* = 0.50) than GSA (*semipartial r* = 0.23). Similar results were previously reported by Phillips et al. (2013) who showed that symptom severity in 82 IBS patients was significantly predicted by the DIF (β = 0.24) and DDF (β = −0.37) factors of the TAS-20, together with gender (β = 0.15), by accounting for 38.5% of the variance of illness severity (*R*^2^ = 0.38; *p* < 0.001). Consistently, a recent follow-up study aimed at investigating whether symptom improvement in 150 IBS patients could be independently predicted by the level of alexithymia and GSA, over and above other clinical and psychological factors (Porcelli et al., 2017). The overtime stability of alexithymia was investigated with hierarchical regression showing that baseline TAS-20 scores significantly predicted TAS-20 scores at follow-up (*R*^2^ = 0.53; *semipartial r* = 0.39; *p* < 0.01). Baseline alexithymia (*Cox & Snell R*^2^ = 0.15; Δ*R*^2^ = 0.10; *p* < 0.001) and gastrointestinal symptoms (*Cox & Snell R*^2^ = 0.01; Δ*R*^2^ = 0.01; *p* < 0.009), but not GSA (*Cox & Snell R*^2^ = 0.05; Δ*R*^2^ = 0.03; *p* < 0.21), significantly and independently predicted also post-treatment improvement status with an overall classification rate of 74%. Moreover, after controlling for co-variables, alexithymia emerged as the unique significant predictor of symptom improvement by adding 17% of explained variance of change in IBS severity symptoms (*R*^2^ = 0.23; Δ*R*^2^ = 0.17; *semipartial r* = −0.41; *p* < 0.001). In other terms, although alexithymia and GSA were closely related to IBS symptoms, only alexithymia was to found to be a stable trait and a stronger predictor of treatment outcome than GSA.

The predictive value of alexithymia in GI symptom persistence was investigated in 52 patients with gallstone disease after laparoscopic cholecystectomy (Porcelli et al., 2007a). After assigning patients to either an post-surgery improved or an unimproved outcome group, the authors showed not only that at baseline the unimproved patients scored significantly higher on the TAS-20 (66.00 ± 7.79) than the improved counterpart (49.32 ± 11.50) (*t* = 5.80, *p* < 0.001, *d* = 1.70), but also that alexithymia and psychological distress significantly predicted improvement of GI symptoms [*R*^2^ = 0.59, *F*_(2, 49)_ = 35.72, *p* < 0.001]. More specifically, TAS-20 total scores added a significant incremental amount of explained variance in predicting GI symptoms [Δ*R*^2^ = 0.38; Δ*F*_(1, 49)_ = 45.17; *p* < 0.001]. A previous study (van Kerkhoven et al., 2006) found similar results in a large sample of 1141 patients referred to endoscopy for upper GI symptoms by showing that high alexithymia patients (21% of the total sample; *n* = 245) reported significantly more GI symptoms (6 ± 4 vs. 5 ± 3; *p* < 0.05) and higher symptom severity (42 ± 34 vs. 34 ± 30; *p* < 0.01) than those with lower levels of alexithymia (79% of patients; *n* = 896). Alexithymia was also related to symptom perception as patients reporting more than 8 GI symptoms had more than 2-fold risk of being classified as alexithymics (*OR* = 2.1; 95% CI = 1.5–2.9). Especially patients with hematemesis (*OR* = 3.7; 95% CI = 1.5–9.2), abdominal pain (*OR* = 1.8; 95% CI = 1.4–2.5), and vomiting (*OR* = 1.9; 95% CI = 1.2–3.0) are indeed frequently identified as subjects with alexithymia. The clinical consequence of alexithymia in FD patients was further underlined in studies reporting that alexithymia and trait anxiety were the main determinants of the mental component of quality of life (Van Oudenhove et al., 2011b; Jones et al., 2013). Specifically, Van Oudenhove et al. (2011b) found that mental quality of life was negatively and significantly associated with alexithymia (Spearman's *rho* = −0.42; *p* < 0.001).

An interesting study investigated the role of alexithymia in comorbid FGID and psychiatric syndromes.

Thirty-eight FGID patients with comorbid psychiatric syndromes recruited in a GI setting were compared with 25 psychiatric outpatients with comorbid FGID recruited in a psychiatric setting (Porcelli et al., 2004). The first group scored significantly higher on the TAS-20 (66.7 ± 11.2) than the latter group (52.7 ± 10.4) (*t* = 4.99, *p* < 0.001), with a prevalence of alexithymia at 81.6% (*n* = 31) compared with a rate of only 28% in the latter group (*n* = 7). Also, TAS-20 total scores independently predicted which subjects belonged to the subgroup of FGID patients with comorbid psychiatric disorders (*OR* = 1.14; 95% CI = 1.03–1.26; *p* = 0.001).

Conflicting results were also found, however. Farinelli et al. (2007) showed that no one of 69 patients with gastroesophageal reflux disease was identified as alexithymic on the basis of the TAS-20 (43.7 ± 9.9). Similar negative results were obtained by Jones et al. (2004) who found that only 12% of 111 FD patients obtained a score of ≥61 on the TAS-20, even though patients with FD scored slightly higher (43 ± 11) than 53 control healthy subjects (38 ± 11) (*p* < 0.005). Similarly, no significant TAS-20 difference was found by Jones et al. (2006) between 74 IBS (42 ± 12) and 48 IBD patients (43 ± 12), even though they were more alexithymic than 55 healthy subjects (38 ± 9) (*p* = 0.005). Same results were reported by other two studies comparing TAS-20 mean scores between 151 FD (47.62 ± 13.06) and 58 non-FD patients (45.62 ± 13.32) (Cohen's *d* = 0.14; *p* = 0.332) (Dibaise et al., 2016), and between 28 patients with FGID (46.16 ± 13.91) and 17 patients reporting GI motility disorders (48.43 ± 17.15) (*z* = −0.63; *p* = 0.527) (Eiroa-Orosa et al., 2015).

#### Neurobiological studies

Only two cross-sectional studies on non-patients from one Japanese group performed an assessment of alexithymia and its neurobiological correlates in GI patients. Kano and her colleagues (Kano et al., 2007, 2015) found that in subjects where visceral sensation was induced by distension of the colon or rectum, the DIF alexithymia factor was significantly associated with subjective symptoms as abdominal distension (*r* = 0.27; *p* < 0.05), abdominal pain (*r* = 0.34; *p* < 0.05), urgency for defecation (*r* = 0.31; *p* < 0.05), perceived stress (*r* = 0.3; *p* < 0.05), sleepiness (*r* = −0.3; *p* < 0.5), and anxiety (*r* = 0.28; *p* < 0.005). They found also that alexithymia significantly and positively correlated with both higher activity in the right insula of the brain (*Z-score* = 4.26; *z* = 4; *p* < 0.001) (which is the primary projection area for visceral afferent information and is critically involved in subjective emotional experience and awareness of the internal bodily state) and orbital gyrus (*Z-score* = 4.29; *z* = −22; *p* < 0.001) (which receives robust sensory inputs and acts as an internal environmental integrator that coordinates behavioral, autonomic, and endocrine responses).

#### Treatment studies

Only one follow-up study by Faramarzi et al. (2013) investigated the effects of psychotherapy on GI symptoms and alexithymia. This study aimed to evaluate the effect of short-term psychodynamic psychotherapy on alexithymia in 24 patients with FD and found that the psychotherapy group significantly improved after treatment for both alexithymia and dyspepsia symptoms (as heartburn/regurgitation, nausea/vomiting, post-prandial fullness/early satiety, bloating, upper or lower abdominal pain) and that the improvement was maintained at 1 and 12 months post-treatment. Specifically, the improvement of alexithymia concerned the TAS-20 total scale [*F*_(3, 45)_ = 25.80; *p* < 0.001] and DIF [*F*_(3, 45)_ = 16.00; *p* < 0.001] and DDF [*F*_(3, 45)_ = 11.70; *p* < 0.001] scores but not the EOT factor.

### IBD and alexithymia

UC and CD, the main forms of IBD, are “complex immunologically mediated diseases that arise due to a dysregulated immune response to commensal flora in a genetically susceptible host” in which “diet, lifestyle and behavior, as well as perturbations of the gut microbiota through use of antibiotics, might also have important roles in disease pathogenesis” (Ananthakrishnan, 2015, p. 206). The clinical course of the disease is characterized by intermittent phases of unforeseeable, intermittent acute relapses and symptom-free periods. Acute episodes are characterized by clinical symptoms of severe abdominal pain, bloody stools, and severe diarrhea and endoscopic and histological signs of inflammation and lesions of the gut mucosa. It is a severe GI disease with likely complications at the intestinal (malnutrition, weight loss, colon cancer, fistulas, intestinal perforation, bowel obstruction) and extra-intestinal (arthritis and ophthalmologic, dermatologic, and urinary complications) manifestations (Kucharzik et al., 2006; Sartor, 2006).

Seven studies on alexithymia with IBD patients had a cross-sectional design (Porcelli et al., 1995; Verissimo et al., 1998, 2000; Boye et al., 2008a,b; Iglesias-Rey et al., 2012; La Barbera et al., 2017), and only 4 were longitudinal (Porcelli et al., 1996; Porcelli and Meyer, 2002; Tibon et al., 2005; Porcelli and Mihura, 2010).

Porcelli et al. (1995) conducted the first research study aimed at evaluating the association between alexithymia and IBD. By comparing 112 IBD patients with 112 matched healthy control subjects, they found a prevalence rate of alexithymia of 35.7% (*n* = 40) in the clinical sample that was higher than the 4.5% (*n* = 5) prevalence in control subjects (χ^2^ = 49.5; *p* < 0.001). The association between TAS-20 and IBD was further confirmed after controlling for the sociodemographic variables of gender, age, and education (χ^2^ = 48.12; *p* < 0.001). Similarly, when comparing a clinical group of 124 patients, of which 74 with IBD, with a control group of 218 medical students, Verissimo et al. (2000) showed that the clinical sample scored significantly higher than controls on the TAS-20 total scale score (56.44 ± 11.66 vs. 48.68 ± 9.15) (*t* = 6.82; *p* < 0.001). Particularly the DIF (20.96 ± 6.88 vs. 17.85 ± 5.30; *t* = 4.66; *p* < 0.001) and EOT (20.82 ± 4.39 vs. 16.18 ± 3.69; *t* = 10.42; *p* < 0.001) factors were significantly higher in clinical sample than controls. In 104 IBD patients investigated longitudinally for 6 months, Porcelli et al. (1996) further supported alexithymia as a stable personality trait through high correlation between baseline and follow-up TAS-20 scores (*r* = 0.95, *p* < 0.001). Another study by Verissimo et al. (1998) confirmed that alexithymia scores were not significantly related to the duration of IBD or the level of disease activity. However, the TAS-20 correlated negatively and significantly with disease-specific quality of life score (*r* = −0.34, *p* < 0.01), particularly with bowel symptoms (*r* = −0.28; *p* < 0.05), systemic symptoms (*r* = −0.37; *p* < 0.01), and emotional functioning (*r* = −0.38; *p* < 0.01). Finally alexithymia (β = −0.27), emotional control (β = 0.26), education (β = −0.7), and socioeconomic index (β = −0.09),played a significant role in predicting levels of quality of life [*R*^2^ = 0.21; *F*_(4, 57)_ = 3.69; *p* < 0.01]. Consistently, Boye et al. (2008b) and La Barbera et al. (2017) examined 109 and 100 IBD patients, respectively, and found that high levels of alexithymia were associated with lower scores of physical, mental and social functions. Specifically, in patients with UC alexithymia negatively and significantly correlated with physical (*r* = −0.33), mental (*r* = −0.49), and social (*r* = −0.30) components of health-related quality of life (Boye et al., 2008b). Similar results have been found by Iglesias-Rey et al. (2012) in a large sample of 484 patients showing that alexithymia, particularly the DIF factor (β = −0.02; *p* ≤ 0.001), represents a significant determinant of impaired health-related quality of life in IBD. More specifically, alexithymia was found as a significant risk factor for poor quality of life (*OR* = 3.34, 95% CI: 1.98–5.65). Consistently, another study by Boye et al. (2008a) found that in 56 UC patients alexithymia negatively and significantly correlated with the emotional dimension of quality of life (*r* = −0.26).

### Liver diseases and alexithymia

Differently from previous studies on FGID and IBD, 8 of the 10 included studies evaluating alexithymia in hepatology had a follow-up study design (Fukunishi et al., 2002, 2003; Nardelli et al., 2013; Palmieri et al., 2013; Porcelli et al., 2014a, 2015; Cozzolongo et al., 2015; Murri et al., 2017). Research investigations focused more on the role that alexithymia might play in the course of disease and medical treatment rather that its prevalence. Prevalence of alexithymia was reported in only 2 studies at 22% (*n* = 100) (Rustgi et al., 2010) and 54% (*n* = 22) (Palmieri et al., 2013).

The first research study investigating alexithymia in hepatology included patients with chronic non-alcoholic liver disease pending for a liver transplantation (Blomhoff and Malt, 1995). The most frequent causes of non-alcoholic liver diseases include primary biliary cirrhosis, primary sclerosing cholangitis, and chronic autoimmune hepatitis, while other potential causes include secondary biliary cirrhosis, CHC, malignant liver cancer, and cirrhosis of medically unknown etiology. In this first study, the authors did not find higher alexithymia levels in their 29 patients who scored in the normal range of TAS-26 (64.0 ± 10.5). Later, Fukunishi et al. (2002) administered the TAS-20 to 31 donor-recipient pairs undergoing living-related liver transplantation and found that pre-transplant alexithymia was significantly related to the manifestation of post-transplant paradoxical psychiatric syndrome (PPS) (χ^2^ = 7.2, *p* = 0.007). The PPS is a reactive psychiatric disorder characterized by core symptoms of prominent conflicts associated with transplantation such as guilt regarding the donor's well-being; situational reactions such as depression, anxiety, somatization; late reaction occurring as a complication following liver transplantation; and favorable medical status of donor and recipient. This psychiatric disorder is defined with the specific term “paradoxical” because such a syndrome occurs despite successful transplantation and without tissue rejection or other medical complication (Fukunishi et al., 2001). The same authors later showed that the severity of alexithymia was significantly higher in 441 recipients before than after transplantation (Fukunishi et al., 2003).

The other liver disease investigated for alexithymia is CHC. It is mainly caused by HCV infection, reaches a worldwide prevalence of about 2% (Shepard et al., 2005), and has been associated to an increased rate of mortality (Lee et al., 2012). HCV is transmitted through exposure to contaminated blood and slowly attacks the liver, advancing to CHC, hepatic cirrhosis or liver cancer (Lauer and Walker, 2001). Before the availability and marketing of new direct anti-viral drugs, standard treatment consisted of a combination therapy with pegylated IFN, a proinflammatory cytokine that modulates the immunologic system, and ribavirin, a nucleoside inhibitor with a broad activity against viral pathogens, for variable periods of 6–12 months. The IFN-based therapy induced patients who were subjectively healthy before starting the treatment to long-lasting (up to 1–2 years after the end of the treatment period) biological adverse events (hematologic, immunologic, infective, cardiovascular diseases, as well as neuropathy, heart, kidney, and lung failures) and subjective somatic symptoms (fatigue, insomnia, pruritus, diarrhea, flu-like syndrome, nausea, and headache). As stated in the Introduction, subjectively reported somatic symptoms are largely overlapped with somatization symptoms. The first study assessing alexithymia in 83 consecutive patients with CHC found that alexithymia was prevalent at 22% (*n* = 18) and significantly associated with the Illness Effects Questionnaire (*r* = 0.40 *p* < 0.001) that measures the individual burden of hepatitis in terms of the subjective appraisal in biologic, emotional, and interpersonal domains (Rustgi et al., 2010). Also after controlling for levels of depression, alexithymia significantly correlated mainly with fatigue (*r* = 0.53; *p* < 0.001) and burden of disease (*r* = 0.41; *p* < 0.001) and less with sleep difficulties (*r* = 0.24; *p* < 0.05). Furthermore, patients designated categorically as alexithymic endorsed significantly greater levels of depression than those that were identified as not alexithymic and alexithymia emerged as the only significant predictor of depression, accounting for 43% of the total variance (*OR* = 1.1; *p* < 0.01).

The predictive value of alexithymia in CHC patients during the standard medical treatment with IFN for 6–12 months that was available at that time was evidenced by Porcelli et al. (2014a). Adjusting for co-factors (lifetime psychopathology, psychological distress, and sustained virological response at follow-up, that is a measure of successful virus eradication), alexithymia and somatizing vulnerability independently predicted IFN-related somatization symptoms while not adverse biological events (as anemia, neutropenia, and thrombocytopenia) during the entire study period, explaining 77– 80% of their variance at both baseline and follow-up evaluations. Specifically, alexithymia (β = 0.26; *p* = 0.001) and somatization (β = 0.66; *p* < 0.001) significantly and independently contributed to predict somatic symptoms at baseline (*R*^2^ = 0.80; *p* < 0.001) and follow-up (β = 0.43; *p* < 0.001, and β = 0.68; *p* < 0.001, respectively; *R*^2^ = 0.77; *p* < 0.001). Consistently, the same research group (Porcelli et al., 2015) found that, after a first follow-up of 3 months (T2), as well as at the end of treatment (T3), alexithymia and a gene variant of the 5-HT1A receptor (HTR1A-G/G) polymorphism (i.e., a functional gene polymorphism in the upstream regulatory region of the serotonin transporter coding sequence associated with major depression), both separately (explained variance: from 20 to 22%; T2: *R*^2^ = 0.22; *F* = 6.83; *p* = 0.01; T3: *R*^2^ = 0.20; *F* = 4.34; *p* = 0.03) and jointly (explained variance: from 14 to16%; T2: *R*^2^ = 0.16; *F* = 7.39; *p* = 0.007; T3: *R*^2^ = 0.14; *F* = 5.53; *p* = 0.03), significantly and independently predicted the development of IFN-induced depression in a sample of 130 HCV patients. Poor quality of life in patients with HCV (*n* = 124) was also independently predicted by alexithymia (at baseline: *semipartial r* = 0.24; at follow-up: *semipartial r* = 0.39) and depression (at baseline: *semipartial r* = 0.24; at follow-up: *semipartial r* = 0.31) before (T0: *R*^2^ = 0.60) and at the 6-month post-IFN treatment follow-up (T3: *R*^2^ = 0.69) (Cozzolongo et al., 2015). Similarly, a previous study on 60 cirrhotic patients found that all domains of health related quality of life, except bodily pain, were significantly impaired in cirrhotic patients presenting with alexithymia (Nardelli et al., 2013). Specifically, the authors showed that alexithymia particularly altered the mental component of health-related quality of life (β = −0.54; *t* = −2.63; *p* = 0.01). Finally, Murri et al. (2017) did not detect a significant role of alexithymia in predicting persistent IFN-related depression but the subsample of 12 HCV patients developing persistent depression reported higher TAS-20 scores (56.7 ± 15.9) than 43 patients without depression (48.7 ± 14.8), although not at a statistically significant level (*t* = 1.62, *p* = 0.11).

## Discussion

The close bidirectional communication between the central and the enteric nervous systems is referred to as the brain-gut axis. Its role is to monitor and coordinate gut functions as well as to link emotional and cognitive centers of the brain with peripheral intestinal mechanisms. Also, the role of gut microbiota is increasingly emerging as one of the major clinical factor that is able to influence health and disease, as well as the subjective perception of visceral sensations. Although this research topic is in its infancy in humans, alterations in bidirectional brain-gut-microbiota interactions are believed to be involved in the pathogenesis of several functional and organic GI disorders, some brain disorders such as autism spectrum disorders, and Parkinson's disease, and psychiatric disturbances such as mood and affect disorders, and chronic pain (Mayer et al., 2015). Given the strict connections between different organs and systems, upstreaming GI sensations and downstreaming emotional and cognitive perceptions are closely intertwined in affecting individual illness behavior (Fava and Sonino, 2017). Based on theoretical assumptions and a wide dataset of research findings (e.g., Taylor, 2000; Taylor and Bagby, 2004, 2012), it is conceivable that alexithymia may be seen as playing an important role in the GI setting.

In the present systematic review, for the first time to our knowledge, the link between alexithymia and GI disorders has been investigated. We expected that alexithymic features would be more prevalent in GI disorders based more on subjective perception than organic biomarkers. Regardless of the etiological nature of illness, we expected also that alexithymia would be more relevant in clinical outcomes related to the subjective dimension of health perception. The main findings of our systematic review are generally consistent with our expectations. Specifically, we have found that:

1. the prevalence of alexithymia was significantly higher in GI patients compared to control subjects and to the estimated prevalence rates in the general population (10–15%; see Mattila et al., 2006; Franz et al., 2008; Tolmunen et al., 2011);
2. alexithymia was higher in patients with FGID than in those with IBD and liver diseases;
3. most significant findings concerned the difficulty identifying and communicating feelings (the DIF and DDF factors of the TAS-20), whereas no significant results emerged generally as for the external-oriented thinking facet of alexithymia;
4. the relevance of alexithymia (i.e., the impact and its main consequences) was significantly more related to somatization, health-related perception, symptom persistence and reporting, and negative treatment outcomes. However, these results should be considered in the light of several limitations of the reviewed studies that will be highlighted later.

### Prevalence rates of alexithymia

Based on the widely used cut-off scores of the TAS-26 and TAS-20, in the GI clinical setting alexithymia has been found consistently highly prevalent in patients with FGID, with rates ranging from 66% (Porcelli et al., 1999) to 87% (Van Oudenhove et al., 2008). The prevalence of alexithymia in IBD patients, though lower than in FGID, is still higher than the general population, at about 30–35% (e.g., Porcelli et al., 1995; Iglesias-Rey et al., 2012). Unlike FGID, however, conflicting results in IBD patients were reported by some investigations showing prevalence at similar level of general population, if not even lower (Jones et al., 2006; Boye et al., 2008a,b). Hence, currently there is no consensus about the prevalence rate of alexithymia in IBD.

Prevalence of alexithymia in hepatology is based on a lower number of studies. Palmieri et al. (2013) found a high prevalence rate of alexithymia in 54% of cirrhotic patients. This frequency of alexithymia is higher than the prevalence rate of 23% reported in the same population of cirrhotic patients by Nardelli et al. (2013), but it is comparable to the prevalence of alexithymia reported in other chronic disease such as obstructive pulmonary disease (Han et al., 2012), essential hypertension (Jula et al., 1999), psoriasis (Sampogna et al., 2017), and type 2 diabetes (Lemche et al., 2014; Avci and Kelleci, 2016).

Overall, in gastroenterology and hepatology the prevalence of alexithymia was higher than the general population and control subjects. Furthermore, alexithymia was found in more than two third of samples of FGID patients. By contrast, in organic disorders like IBD and liver disease, alexithymia was prevalent only in one third to one half of samples, similar to other non-GI chronic diseases.

### Alexithymia and clinical outcomes

#### Associations with health-related issues

In line with the view of alexithymia as a personality dimension associated with affective dysregulation, several studies have found consistently that alexithymic traits negatively influence a series of clinical outcomes, that are strictly related to the subjective perception of health. Specifically, the following were the main results in this regard: (1) in patients with FGID (Arun, 1998; Jones et al., 2004, 2006) and also in those with non-alcoholic liver disease (Blomhoff and Malt, 1995) higher scores of alexithymia were directly associated with higher levels of negative emotional traits such as neuroticism, rigidity, introversion, and perfectionism; (2) there was a significant relationship between alexithymia and depressive symptoms in patients with FGID and peptic ulcer (Faramarzi et al., 2014), as well as in those with HCV and cirrhosis (Rustgi et al., 2010; Nardelli et al., 2013; Murri et al., 2017); (3) alexithymia correlated significantly with insecure attachment relations in patients with IBD (Bengtsson et al., 2013) and gastroesophageal reflux disorder (Farinelli et al., 2007); (4) alexithymia was significantly associated with lower quality of life and impaired psychosocial functioning in patients with HCV (Cozzolongo et al., 2015) and liver carcinoma (Palmieri et al., 2013), IBD (Verissimo et al., 1998; Iglesias-Rey et al., 2012; La Barbera et al., 2017), and FGID as well (Portincasa et al., 2003; Van Oudenhove et al., 2011b; Jones et al., 2013).

#### Symptom perception and persistence

Compared to patients with lower levels of alexithymia, those characterized by higher alexithymia reported more severe GI symptoms. In particular, patients with IBS perceived higher levels of symptom severity even when controlled for GI-specific anxiety (Porcelli et al., 2014b). Similarly, patients with gallstone disease reported persistence of dyspeptic symptoms 1 year after cholecystectomy (Porcelli et al., 2007a), and those with HCV infection had higher somatic symptoms during antiviral treatment (Porcelli et al., 2014a) if scoring higher on alexithymia. However, results are not consistent across investigations and conflicting findings on the association of alexithymia with symptom severity have been reported also (Van Oudenhove et al., 2008; Jones et al., 2013; Dibaise et al., 2016).

#### Treatment outcomes

Some studies investigated the role of alexithymia in explaining treatment outcomes. After 6 months of enhanced treatment with medical and psychological interventions, alexithymia showed to be a stable trait over time that influences negatively treatment outcomes, even after controlling for baseline GI symptoms, depression, and anxiety (Porcelli et al., 2003). In IBS patients, alexithymia was found to be a stronger predictor of symptom severity and negative outcomes following enhanced usual care compared to other clinically relevant constructs as visceral sensitivity (Porcelli et al., 2017). Finally, in a randomized controlled trial, significant improvement of alexithymia and GI symptoms 6 months after short-term psychodynamic psychotherapy (combined with standard medical treatment) was found in FD patients compared to those who received as-usual medical care only (Faramarzi et al., 2013).

#### Associations with clinical issues in liver disease

Except for findings on prevalence reported above (e.g., Nardelli et al., 2013; Palmieri et al., 2013), few studies have investigated how much alexithymia may influence clinical issues in hepatology. In patients with CHC undergoing antiviral treatment, alexithymia (particularly the facets of difficulty identifying and describing feelings), alone or together with tendency to somatization, was more prominent in explaining subjective somatic symptoms (i.e., fatigue, insomnia, pruritus, diarrhea, flu-like syndrome, nausea, and headache), but not the biological adverse events (i.e., anemia, neutropenia, and thrombocytopenia) (Porcelli et al., 2014a). Alexithymia contributed also to impair quality of life even when lifetime mood disorders were controlled for (Cozzolongo et al., 2015), at the entry of the study and after 6 months from the termination of treatment with IFN. Of interest, in these patients alexithymia and the presence of the G-variant polymorphism of the HTR1A, a promoter region of the serotonin receptor gene, explained higher vulnerability to depressive symptoms during the antiviral treatment (Porcelli et al., 2015).

Findings on the role of alexithymia in treatment outcomes and liver diseases should be however viewed with caution, as they come from a single research group and therefore cannot be compared with independent research investigations.

### Potential mechanisms of alexithymia

Alexithymia has been extensively investigated in the last 30 years. There is indeed a lot of evidences that alexithymia is associated with personality traits affecting mental and somatic health. Its several clinical consequences are neuroticism, harm avoidance, low openness to experience and psychological mindedness (Taylor, 2000), insecure attachment (Taylor et al., 2014), implicit mechanisms of emotional recognition in interpersonal relations (Donges and Suslow, 2017), higher somatic symptom reporting (Mattila et al., 2008) and perception (Lumley et al., 1996; Porcelli et al., 2007b), altered immune and inflammation status (Honkalampi et al., 2011), persistence of somatic symptoms and negative outcomes of medical treatments (McIntosh et al., 2014; Baudic et al., 2016; Saariaho et al., 2017), increased genetic susceptibility (Terock et al., 2018), and higher mortality risk over long periods (Tolmunen et al., 2010). In the clinical link between alexithymia and GI disorders, it is not easy to disentangle which co-variate is associated to which. Data suggest that alexithymia is prevalent in high rate in GI disorders and is particularly relevant in modulating the individual perception of symptoms, even after as-usual medical care and independently of the nature of the GI condition.

Several factors may explain the association of alexithymia with GI symptoms and health-related issues.

One is represented by the proneness of alexithymic individuals to experience and to report more functional somatic symptoms because of their tendency to amplify, selectively focus on, and misinterpret the somatic sensations that accompany states of emotional arousal, as well as other normal bodily sensations (Nakao et al., 2002; Jones et al., 2004; Van Oudenhove et al., 2016). It has been hypothesized that the diminished ability of alexithymic individuals to experience emotions as conscious feelings may lead to a significantly amplification of the somatic sensations accompanying emotional arousal (Barsky and Klerman, 1983; Lane and Schwartz, 1987; Lane and Garfield, 2005). The higher prevalence of alexithymia among patients with functional illnesses such as IBS and FD than in those with organic diseases such as IBD and HCV is thought to be due to this perceptual mechanism.

Another pathway is represented by the bidirectional brain-gut axis that allows GI symptoms to be generated not only by motility disturbances or alterations in sensory inputs originating in the gut, but also by unregulated states of emotional arousal that, when mutually interact with somatic sensations of the gut and with other psychological factors, lead individuals to develop visceral hypersensitivity (Mayer, 2011). IBS patients have been found to have lower thresholds for visceral sensations and pain perception, and central alteration of abnormal pain evaluation. This is thought to be attributable not only to bottom-up signals from the gut to the brain, but also to complementary top-down affective disturbances clinically related to functional alterations of prefrontal and cingulate regions (Elsenbruch, 2011; Stabell et al., 2013). Alexithymia, as an individual deficit characterized by difficulties with emotion regulation and reduced emotional awareness, may determine a significantly lower threshold to somatic sensations arising from the gut. A large body of literature repeatedly confirmed the association between alexithymia and increased severity of subjective somatic symptom reporting (Kooiman et al., 2000; Lumley et al., 2007; Tolmunen et al., 2010; Stabell et al., 2013).

Furthermore, alexithymic individuals reported higher activation of processes leading to amplification of visceral states at multiple levels. They have been found to exhibit higher resting or tonic sympathetic activity (Stone and Nielson, 2001; Alkan Härtwig et al., 2013) as evidenced by: (1) altered interoceptive awareness (Herbert et al., 2011); (2) higher activation of pain-related brain areas (e.g., insula, pregenual anterior cingulate cortex, and brainstem); (3) lower awareness of the internal body states (Kano et al., 2007, 2015); (4) higher pro-inflammatory activity (i.e., higher C-reactive protein and altered balance of pro- and anti-inflammatory cytokines) (De Berardis et al., 2008; Guilbaud et al., 2009; Honkalampi et al., 2011). Overall, these findings suggest that difficulties with emotion regulation through reduced emotional awareness via alexithymia may negatively impact the subjective pain experience and increase the likelihood of reporting high somatic symptoms.

A third pathway may be identified in the association with illness behavior (Sirri et al., 2013). The construct of illness behavior refers to the varying ways individuals respond to bodily sensations, monitor internal states, interpret somatic symptoms, and use health care services (Sirri et al., 2013). Alexithymia may prompt a maladaptive and unhealthy lifestyle serving as modulator of unregulated emotional arousal (Kauhanen et al., 1992; Pinaquy et al., 2003). Unhealthy behaviors such as poor nutritional consumption, poor eating behavior (e.g., fast eating and binging), alcohol and drug use, sedentary lifestyle may be conceived as maladaptive coping strategies and efforts that individuals with alexithymia use to self-regulate distressing emotions that they cannot process at a cognitive level (Kauhanen et al., 1992; Pinaquy et al., 2003). Various combinations of somatic amplification, lower threshold to pain, unhealthy lifestyle, and abnormal illness behavior might be the underlying mechanisms through which alexithymia, as a predisposing or mediating factor, is linked to symptom formation and poor treatment outcomes in FGID and to overlapping somatization symptoms in organic conditions such as IBD and HCV.

The likely role of alexithymia in disorders of the GI system can be viewed within the framework of contemporary theoretical models of the mind-body-brain relations. For example, Damasio (1999) speculated on two different neural pathways of “body loop” and “as-if body loop.” In the first model, physiological inputs are relayed into the brain, where they are later transformed in emotions that form somatic markers. The enacted somatic states can then activate regions involved in body mapping, trigger somatic states thus increasing or decreasing thresholds for subsequent somatic states. These somatic states can simultaneously activate working memory thus strengthening or weakening a particular representation in the brain. In the second pathway, instead of having somatic states expressed in the body, cognitive representations of the emotions (e.g., imagining an unpleasant situation “as-if” one were there) may activate representations of somatic states in the brain thus inducing changes in neurotransmitter release but without re-enacting the body. The individual brain might therefore anticipate expected bodily changes without being directly elicited by a sensory stimulus and allows the individual to respond faster to external stimuli without waiting for an event to actually occur. Damasio's “as-if body loop” may serve either the adaptive purpose of planning future behavior in advance and the maladaptive pattern of re-mapping bodily states as perception of somatic illness, particularly if feelings are defectively linked to somatic sensations of autonomic arousal, according to the alexithymia construct.

Another theoretical model that is gaining interest recently conceptualizes somatization as a disorder of perception (Wiech, 2016; Henningsen et al., 2018). In this model, perception of bodily states, including inputs from the enteric nervous system, is co-determined by top-down expectations generated by the central nervous system that is continuously building probabilistic predictions of its environment. Such predictions include the mapping of bodily states, like a coding machine. Higher perception of GI symptoms may arise when downstreaming predictions of distress meet with rougher upstreaming inputs from the gut. Symptoms may be therefore based on this sort of mismatch between expectations and sensations. Several predisposing, triggering, and maintaining factors modulate how individuals would perceive their health status, report the outcomes of their illness, and shape their illness behavior, from spontaneous remission of mild symptoms to denial of being ill to amplification of symptoms to chronicity of disease. Environmental and psychosocial factors may act as triggering (e.g., a viral infection as in post-infective IBS or a stressful event as in many acute episodes of FGID) or maintaining (e.g., frustration stemming from the burden of antiviral treatment in HCV or a persistent low-grade inflammation in post-relapsing phases of IBD). Predisposing or vulnerability factors are powerful variables in paving the way toward different individual pathways of illness behavior. Also, the cultural background (Kirmayer and Sartorius, 2007), childhood traumas, attachment patterns (Maunder et al., 2017), and epigenetic mechanisms play a significant role in this regard (Turecki and Meaney, 2014).

Alexithymia may be considered as one of predisposing psychological factors as well as one of the determinants involved in the Damasio's “as-if body loop.” Deficits in processing and regulating affects may constitute indeed a strong personality-related vulnerability aspect in disrupting the matching process between central expectations and peripheral sensations generating emotions. This may explain the high prevalence rate of alexithymia in medical and psychiatric disorders (Taylor et al., 1997) as well as the overlap between alexithymia and mood, anxiety, and somatization symptoms (Porcelli et al., 2013).

### Limitations

Caution should be expressed when interpreting the findings of this systematic review because of the limits of the reviewed studies. Overall, studies prevalently involved patients with FGID (27 of the 48 reviewed studies), samples sizes were generally small (under 100 participants), adopted a cross-sectional design (30 studies), and used only one method for assessing alexithymia.

Studies with small sample size constitute a strong limitation for the generalization of results. In the GI setting, as well as in other medical settings, and particularly in patients with functional somatic disorders, patients recruited from secondary and tertiary care centers have been shown to have moderate to severe illness severity (Drossman, 2016). In these patients, representing the most severe end of severity continuum, high level of alexithymia may co-occur—and confounded with—higher psychological distress, psychiatric comorbidity, and abnormal illness behavior. However, the higher prevalence rate of alexithymia in FGID than IBD and liver disease patients than the estimated rate in the general population might be biased by these confounding factors.

Cross-sectional studies do not allow to establish direction of causality or the overtime stability of alexithymia. Patients recruited in secondary and tertiary care settings generally show longer duration of illness, previous tentative treatments, and therefore may be frustrated by the unwanted effects of therapy. Usually these patients are considered by their physicians as “difficult patients,” thus limiting the benefits of an effective doctor-patient relationship (Aronson, 2013). Alexithymic features may therefore be a consequence rather than a predisposing factor of the illness status. Furthermore, some studies showed the relative stability of alexithymia within the context of change of symptoms following treatment both in FGID (Porcelli et al., 2003, 2017) and cancer patients (Luminet et al., 2007; Porcelli et al., 2011), but the cross-sectional design of most reviewed studies does not allow to infer to which extent trait alexithymia may predict the course of illness over time.

Finally, as previously stated in the section of introduction, we selected studies using the Toronto scales. No study assessed alexithymia in a multi-method approach and the TAS was the only used measure. Even though the TAS has shown strong psychometric properties, the results from these investigations are limited by the limits of the TAS (reliance on self-report and ability to self-awareness, and missed core aspects of the construct from the scale contents).

Further studies would be needed for further ascertaining the role played by alexithymia in GI disorders. Sounder investigations should include longitudinal studies on larger samples, and better defined inclusion criteria as baseline illness severity and adjustment for primary vs. secondary/tertiary health care settings. Furthermore, the predisposing or mediating role of alexithymia should be investigated at the light of other likely co-determinants of GI conditions such as the influence of gut microbiota and immune inflammatory processes, as well as lifestyle factors like diet and exercise. Future randomized controlled trials should investigate whether interventions aiming to reduce both alexithymia and symptoms are equally effective on both outcomes and if the improvement of symptoms parallels the improvement of alexithymia, by evaluating also which of the two clinical factors is a stronger predictor of the other.

## Conclusion

This systematic review suggests that alexithymia is largely present in patients with functional disorder of the GI tract in two third or more patients, potentially reinforcing symptom persistence and reducing the positive effects of standard medical treatments. Furthermore, alexithymia is present in about one third of patients with inflammatory chronic disease of the gut, as well as in patients with liver disorders. Alexithymia is associated with several psychological factors increasing the subjective burden of disease and impairing quality of life.

Clinicians should be alerted to take alexithymia into serious account when evaluating, managing, and planning interventions with GI patients. Even with some limitations in mind, the assessment of alexithymia in these patients during all stages of patient management, from diagnosis to treatment outcome, is recommended by using the TAS-20 in combination with several validated instruments (self-report scales, interviews, and personality tests), according to the clinical and research needs. Also, since some intervention trials showed that reducing alexithymia significantly contribute to ameliorate symptoms in patients with cancer-related pain (Tulipani et al., 2010), FGID (Faramarzi et al., 2012; Porcelli et al., 2017), and multisomatoform symptoms (Probst et al., 2017), clinicians are strongly suggested to treat alexithymia as a solid contribution to improve the clinical condition of their patients. Even if not directly associated with positive symptom change, reduction of alexithymia may greatly improve the capacity to recognize one's feelings and to communicate them to others, thus contributing to positive clinical outcomes by enhancing psychosocial functioning, affective regulation (Cameron et al., 2014), and psychological well-being.

## Author contributions

Both of the authors made substantial contributions to the work and approved it for publication. Specifically, DC contributed to the acquisition, qualitative analysis and synthesis of data by drafting the first version of the manuscript. PP contributed to the conception and design of the systematic review and revised the manuscript for intellectual content.

### Conflict of interest statement

The authors declare that the research was conducted in the absence of any commercial or financial relationships that could be construed as a potential conflict of interest.
